# Characteristics of Indigenous primary health care service delivery models: a systematic scoping review

**DOI:** 10.1186/s12992-018-0332-2

**Published:** 2018-01-25

**Authors:** Stephen G. Harfield, Carol Davy, Alexa McArthur, Zachary Munn, Alex Brown, Ngiare Brown

**Affiliations:** 1 Wardliparingga Aboriginal Health Research Unit, South Australian Health and Medical Research Institute, Adelaide, South Australia Australia; 2 School of Public Health, Faculty of Health Sciences, The University of Adelaide, Adelaide, South Australia Australia; 3 Joanna Briggs Institute, Faculty of Health Sciences, The University of Adelaide, Adelaide, South Australia Australia; 4 Sansom Institute for Health Research, University of South Australia, Adelaide, South Australia Australia; 5 School of Education and School of Medicine, University of Wollongong, Wollongong, NSW Australia

**Keywords:** Primary health care, Models of care, Service delivery, Indigenous, Aboriginal and Torres Strait Islander, American Indian and Alaska Native

## Abstract

**Background:**

Indigenous populations have poorer health outcomes compared to their non-Indigenous counterparts. The evolution of Indigenous primary health care services arose from mainstream health services being unable to adequately meet the needs of Indigenous communities and Indigenous peoples often being excluded and marginalised from mainstream health services. Part of the solution has been to establish Indigenous specific primary health care services, for and managed by Indigenous peoples. There are a number of reasons why Indigenous primary health care services are more likely than mainstream services to improve the health of Indigenous communities. Their success is partly due to the fact that they often provide comprehensive programs that incorporate treatment and management, prevention and health promotion, as well as addressing the social determinants of health. However, there are gaps in the evidence base including the characteristics that contribute to the success of Indigenous primary health care services in providing comprehensive primary health care. This systematic scoping review aims to identify the characteristics of Indigenous primary health care service delivery models.

**Method:**

This systematic scoping review was led by an Aboriginal researcher, using the Joanna Briggs Institute Scoping Review Methodology. All published peer-reviewed and grey literature indexed in PubMed, EBSCO CINAHL, Embase, Informit, Mednar, and Trove databases from September 1978 to May 2015 were reviewed for inclusion. Studies were included if they describe the characteristics of service delivery models implemented within an Indigenous primary health care service. Sixty-two studies met the inclusion criteria. Data were extracted and then thematically analysed to identify the characteristics of Indigenous PHC service delivery models.

**Results:**

Culture was the most prominent characteristic underpinning all of the other seven characteristics which were identified – accessible health services, community participation, continuous quality improvement, culturally appropriate and skilled workforce, flexible approach to care, holistic health care, and self-determination and empowerment.

**Conclusion:**

While the eight characteristics were clearly distinguishable within the review, the interdependence between each characteristic was also evident. These findings were used to develop a new Indigenous PHC Service Delivery Model, which clearly demonstrates some of the unique characteristics of Indigenous specific models.

**Electronic supplementary material:**

The online version of this article (10.1186/s12992-018-0332-2) contains supplementary material, which is available to authorized users.

## Background

Indigenous populations have poorer health outcomes compared to their non-Indigenous counterparts [1]. The experience of colonisation, and the long-term effects of being colonised, has caused inequalities in Indigenous health status, including physical, social, emotional, and mental health and wellbeing [2]. For example, in 2012 the gap in life expectancy between Aboriginal and Torres Strait Islander Australians and non-Indigenous Australians was 10 years [3]. Similar gaps in life expectancy exist in New Zealand [4], Canada [5] and the United States [6].

The evolution of Indigenous primary health care (PHC) services arose from the inability of mainstream health services to adequately meet the needs of Indigenous communities [3, 7, 8]. It was also a response to the reality that Indigenous peoples were often excluded and marginalised from mainstream health services [9]. Part of the solution has been to establish Indigenous specific PHC services, for and managed by Indigenous peoples.

In Australia, the first Aboriginal PHC service was established in 1971 [9] and there are now over 150 Aboriginal Community Controlled Health services across the country [10]. In New Zealand, health reform in the early 1990’s led to the development of Māori health providers. This has resulted in a combination of national and locally controlled Māori led initiatives that are committed to improving Māori health [7]. In Canada, the enactment of the Health Transfer Policy in the late 1980’s initiated the transfer of existing community-based and regional health services into First Nation and Inuit control [11, 12], and more recently the establishment of First Nations and Inuit Health Authorities [13]. In the United States, the provision of health services for American Indians and Alaska Natives began as early as the nineteenth Century and continued through the 1930’s, 1950’s and 1970’s with a number of policy reforms, culminating in what is now known as the Indian Health Services [14–16].

There are a number of reasons why Indigenous PHC services are more likely than mainstream services to improve the health of Indigenous communities. One of the primary reasons is that Indigenous PHC services are often controlled by their local communities [7, 13, 14, 17] and therefore are underpinned by the values and principles of the communities they serve [18]. Their success is also due to the fact that they often provide comprehensive programs that incorporate treatment and management, prevention and health promotion, as well as addressing the social determinants of health [14].

Despite their success, there are gaps in the evidence base including the characteristics that contribute to the success of Indigenous PHC services in providing comprehensive PHC. This systematic scoping review sought to address this gap by identifying the characteristics (values, principles, and components) of Indigenous PHC service delivery models.

## Method

In 2015, the Leadership Group as part of The Centre of Research Excellence in Aboriginal Chronic Disease Knowledge Translation and Exchange, identified the need to document the characteristics of Indigenous PHC service delivery models. Guided by the Leadership Group, a review team was formed comprising one Aboriginal [SH] and three non-Indigenous researchers [CD, AM, ZM]. A key feature of this review was the combination of perspective and skills that the Leadership Group and the researchers brought to the project. This included expertise in systematic and scoping reviews as well as an understanding of Indigenous beliefs, values and experiences.

A scoping review methodology was chosen, as it is the most appropriate methodology for synthesising a body of evidence that has yet to be comprehensively reviewed [19]. Additionally, scoping review methodology is acknowledged as appropriate method to identify concepts or characteristics in the literature [19], such as the characteristics of Indigenous PHC service delivery models. This systematic scoping review followed the Joanna Briggs Institute Scoping Review Methodology [20]. The review team developed and published a protocol prior to commencing the systematic scoping review [21], that outlined the intended approach and method, which is summarised below.

### Inclusion criteria

Concept – the characteristics (values, principles, and components) of service delivery models implemented within an Indigenous PHC service.

Context – PHC services that provided care predominantly for Indigenous peoples.

Indigenous peoples were defined as:

*Indigenous populations are communities that live within, or are attached to, geographically distinct traditional habitats or ancestral territories, and who identify themselves as being part of a distinct cultural group, descended from groups present in the area before modern states were created and current borders defined. They generally maintain cultural and social identities, and social, economic, cultural and political institutions, separate from the mainstream or dominant society or culture* ([22](para. 1)).

Primary health care was defined as:

*socially appropriate, universally accessible, scientifically sound first level care provided by health services and systems with a suitably trained workforce comprised of multi-disciplinary teams supported by integrated referral systems in a way that: gives priority to those most in need and addresses health inequalities; maximises community and individual self-reliance, participation and control; and involves collaboration and partnership with other sectors to promote public health. Comprehensive primary health care includes health promotion, illness prevention, treatment and care of the sick, community development, and advocacy and rehabilitation* ([23](para. 3)).

The above definitions ensured all reviewers shared the same understanding of Indigenous and PHC and all included studies met the inclusion criteria.

### Types of studies

All study types and methods including grey (unpublished) literature published in English between September 1978 and May 2015 were considered. Given that the concept of PHC was broadly adopted in September 1978 [24], papers prior to this date were excluded.

### Search terms

Aboriginal OR Aborigine OR Indigenous OR First Nation OR Maori OR Inuit OR American Indian OR Alaskan Native OR Native Hawaiian AND primary health care OR comprehensive primary health care OR medical service OR health service OR community care OR community health care AND model.

### Search strategy

An initial search of *PubMed* was conducted to identify text words contained in the title and abstract as well as any index terms that could be used as alternate search terms. A second, more detailed search was then undertaken using the identified search terms across *PubMed*, *EBSCO CINAHL*, *Embase*, *Informit*, *Mednar*, and *Trove*. The detailed search strategy used for *PubMed*, which is the basis for all other databases searched can be found in Additional file 1. The reference list of all identified studies were also hand-searched for additional studies which met the inclusion criteria. As a final step, a post was placed on *ResearchGate* to identify any additional literature (particularly grey literature) which may not have been widely available through conventional databases.

### Study selection

The selection of studies was performed by four of the authors [SH, CD, AM, ZM] over two stages – title and abstract review; and full text review, against the inclusion criteria. During the two stages of study selection, author one [SH] reviewed all studies and this was checked by either one of the other three authors [CD, AM, ZM]. Any disagreements were discussed and resolved by authors.

### Charting of data

All papers were imported into QSR International’s NVivo 10 software [25] for extraction of reported characteristics. This analysis was initially conducted independently by the four authors [SH, CD, AM, ZM]; [SH] checked the data extraction and analysis of the three other authors [CD, AM, ZM], while a combination of the other authors checked the data extraction and analysis initially conducted by [SH]. Any disagreements were discussed and resolved between the four authors. Thematic analysis was used to group the extracted findings into characteristics. Findings were reviewed by members of the Leadership Group on two separate occasions during the synthesis process in order to provide an Aboriginal and Torres Strait Islander perspective on their validity.

## Results

**Fig. 1:**
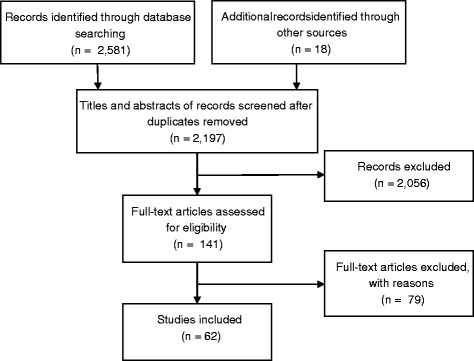
Flow chart of study selection for scoping review process

The original search identified 2599 studies (Fig. 1), from which 402 duplicates were removed, leaving 2197 studies for screening of title and abstract against the inclusion criteria. From this we retrieved 141 studies for full text review, of these 62 met the inclusion criteria (Additional file 2). Study selection follows the PRISMA reporting guidelines for study selection [26].

### Description of studies

The majority of studies included in the review were conducted in Australia [27–58], 18 studies were conducted in the United States [59–76], five in Canada [77–81], four studies in New Zealand [82–85] and one study each in Papua New Guinea [86], Mexico [87] and Peru [88]. Of the studies included, the majority of service delivery models focused on PHC service delivery more generally [29–32, 34, 36, 40–42, 49, 52–54, 56, 58, 61, 64, 66, 67, 69, 71, 73, 75, 76, 81, 82, 84–86]; while the others studies focused on specific areas such as women’s, maternal and infant health [27, 45, 47, 57, 60, 78, 79, 87, 88]; mental health [70, 78, 80]; oral health [33, 39, 65]; eye health [62, 77]; adult health [38]; prevention and health promotion [63, 72, 74]; public and environmental health [59]; homecare [68]; prison health [50, 51, 55]; asthma [35, 37]; diabetes [83]; alcohol and other drugs [46]; medicine access [89]; and continuous quality improvement [28, 43].

### Characteristics

Of the included studies, eight characteristics of Indigenous PHC service delivery models were identified – accessible health services, community participation, continuous quality improvement, culturally appropriate and skilled workforce, culture, flexible approach to care, holistic health care, and self-determination and empowerment. These characteristics underpin many of the service delivery models in this review.

While culture was initially thought to be one of the eight characteristics identified, it became evident through the process of thematic analysis, that it played a central role and was embedded throughout all Indigenous PHC service delivery models. Although the studies did not provide a definition of culture in the context of Indigenous PHC services, they did however demonstrate how aspects of culture were embedded within services and how culture is respected.

Pivotal strategies for embedding culture included the incorporation of local Indigenous cultural values [30, 54, 69, 73, 82]; customs and beliefs [38, 53, 75, 77, 79, 80, 82, 85, 88], as well as traditional healing and practices [36, 77, 79] into the service delivery model. Focusing on the needs of the individual and on the health and wellbeing of their families and communities [33, 34, 85]; respecting women’s and men’s cultural needs [38, 41, 45, 72, 87], such as women only discussing women’s health business with other women [45] or gender specific services and programs [41, 72, 87]; and ensuring the local communities were engaged with [40, 85] and in control of, Indigenous health services, [52, 79, 85] were ways in which culture was embedded into service delivery models and ensured the delivery of culturally appropriate care and made services more acceptable to Indigenous communities. Other practical examples of how culturally appropriate service delivery was achieved, included creating welcoming [41] and comfortable spaces [72], and family-friendly environments [48, 85], through to for example, the use of Indigenous artwork and Indigenous signage [27], and developing culturally appropriate prevention and health promotion resources [37, 50, 63, 72].

Culture was strengthened in many instances by ensuring local languages were spoken within the service [38, 41, 61, 72, 77, 80, 87, 88]. This was often achieved through the employment of local Indigenous staff who also acted as interpreters for non-Indigenous health staff [81]. The employment of local Indigenous staff also incorporated aspects of cultural mentoring [38, 45, 81] ensuring non-Indigenous staff were culturally competent [45, 84] and aware of local protocols and values [58]. This contributed to ensuring cultural safety, a concept which extended beyond being simply aware of cultural differences to incorporating a deeper level of interaction and thoughtful practice, as defined by those who receive services [38, 47, 48, 50, 52, 56, 66, 79].

The other seven characteristics of Indigenous PHC service delivery models are described in Table 1.

**Table 1:** Characteristics of indigenous PHC service delivery models

**Accessible Health Services** • providing **affordable** health care [61, 87] at either no cost or low cost [85]. • ensuring a broad range of services are **available** [50], in a variety of locations and settings [29, 30, 37, 41, 47, 49, 56, 71–73, 78, 80–82, 84, 87], including increased opening hours, walk in appointments [41, 47, 52, 60, 68, 76] and transport [47, 60, 72]. • delivering **acceptable** care which focused on building trust with communities [87] ensuring patients felt supported [38] providing assurances in relation to privacy and confidentiality [38, 80], and implementing services underpinned by cultural respect, social justice and equality [47]. • ensuring **awareness** in the sense that communities know the service exists by providing outreach and mobile services [82], participating in community events and holding screening days [38], encouraging patients to share their positive experiences and promote the service to others [71].
**Community Participation** • ensuring **Indigenous ownership** of health services which enables Indigenous peoples to own and manage their health service [85], and ensures the service is accountable to the community [54]. • establishing **Indigenous governance** [30–32, 34, 35, 40, 60, 61, 64, 70, 82] including members from local community on governing boards, in order to encourage community involvement and ownership, while at the same time building capacity within local communities [41, 50, 53]. • facilitating **community consultation, engagement and collaboration** in order to establish a strong relationship with the community [32, 84] and facilitate sharing of information [83, 88] ensuring that programs were culturally appropriate, accessible, engaging and empowering, and designed to take account of the local context and needs [34, 47, 54, 58, 74, 80, 82, 87]. • respecting the **role and status of elders** [36] and facilitating their involvement in the work and governance of services [45, 63, 66].
**Continuous Quality Improvement** • collecting and utilising **data** not simply to improve health outcomes but also meet the needs of each community by undertaking program evaluation [29], participating in quality improvement initiatives [28], reporting on performance [43], identifying clients who require a specific service, follow-up visits and client tracking [62], service planning and implementation [42, 64, 82], and service impact [42]. • **evaluating services** to measure health benefits for the community [54], assessing economic outcomes, baseline demographics in relation to increased service utilisation, health assessments and chronic disease care plans [42]. • undertaking **research** to strengthen health systems in order to meet the needs of the community with an emphasis on translating research findings into practice [28]. • establishing **quality improvement** processes with a focus on chronic conditions, monitoring health programs as well as management and follow-up care [43, 62], and community involvement in developing indicators [64], that also focus on cultural aspects of care, traditional approaches and receiving care in language [80].
**Culturally Appropriate and Skilled Workforce** • **employing a range of skilled staff** both health and non-health personnel who are able to meet the needs of the local community [30, 36, 41, 49, 50]. • establishing an **Indigenous workforce** comprising Indigenous Health Workers, mental health workers, social workers, nurses, doctors, administration staff, managers and traditional healers, all who are central to the delivery of services, providing a diverse range of care [37, 38, 41, 47, 50, 78, 81, 88], and within some service models, conducting the majority of the clinical work with clients [31, 38]. • recognising that **Indigenous staff often have responsibilities and obligations** in relation to family and community, which were often conducted within the health service [54], such as interpreting and acting as mediators [78]. • providing **training and development opportunities for all staff** [30, 32, 41, 47, 49, 50, 61, 64, 70, 74, 88], such as cultural awareness training for non-Indigenous staff [38, 58, 81], and comprehensive training for staff working in remote areas or in isolation who require additional skills and knowledge to deal with an array of more complex needs [61]. • training **Indigenous Health Workers** [30, 32, 49, 50, 61, 70] as a capacity building exercise [88], including more specialised health care such as dental [33, 39] or maternal health [47]. • recognising **the need to build and grow the Indigenous workforce of the future** by establishing long term strategies to mentor and recruit Indigenous students into health careers [80], leading to Indigenous staff going on to further training or study [32].
**Flexible Approaches to Care** • **tailoring approaches** to identify [36, 50, 55, 66, 72] and meet the needs of the local community [29, 38, 41, 47, 48, 50, 52, 54, 58, 64, 69, 70, 83, 87], and delivering a range of services [29], that are relevant, culturally appropriate and effective [36, 82]. • **integrating health care** services [38, 69, 76, 78, 80, 84], with a multidisciplinary team approach [34, 49, 62, 67–69, 76], case management [33, 46, 67, 72, 76, 80], and continuity of care [47, 48, 53, 56, 67, 68, 81, 84]. • **partnering and linking with other services** to promote integration and cooperation between all support services, providing holistic care through a social view of health [39, 41, 47, 51, 80].
**Holistic Health Care** • providing **comprehensive primary health care**, which is **holistic** [32, 34, 37, 80, 82], supports the health and wellbeing of not only the individual but also their family and community [34, 49–51, 54, 66, 69, 73, 85], and includes mental, emotional and spiritual needs alongside physical wellbeing [57, 60, 80, 84]. • offering **a diverse range of services** to clients [57], such as prevention and health promotion through to chronic disease care [29, 31, 34, 41, 53, 57, 62, 71], maternal and child health [34, 41, 49, 56, 57, 59, 82], oral health [39, 42, 50, 53, 57, 64, 65, 82], ear health [53, 56, 64] sexual health [53], mental and social health [34, 41, 56, 57, 59, 78], alcohol and other drugs treatment [50, 53, 82], pharmaceutical services [44, 56, 69, 78, 80], aged care [29, 36, 41] and disability services [41, 82]. • including **prevention and health promotion** initiatives developed and tailored to the needs of the local community, including general screening programs [29, 38, 63, 72, 77, 82] healthy lifestyles programs [56] needle exchange programs [29, 33, 56, 82], women’s and men’s health programs [29, 34], healthy eating, exercise and smoking cessation programs [31, 32, 49, 52, 56, 71, 77], oral health [65], injury prevention [74], and supporting people to manage their own health [71]. • improving **health literacy** particularly in relation to early warning signs of suicide [70], increasing HIV/AIDs awareness [32], providing information about the harm that comes from alcohol, tobacco and other drugs [73], understanding food labelling [83], maintaining health and ensuring that people could detect early warning signs and understanding when to seek health care advice [71, 83]. • providing **traditional healing** as one option within the health service [36, 52, 59, 63, 64, 70, 72, 73, 77, 79–82, 84–88]. • **advocating for clients** [29, 52], especially in relation to clients moving between other primary, secondary, tertiary, and essential non-health services [60, 84]. • engaging with the **social determinants of health** by supporting clients in accessing housing, employment, education, social security payments, and supporting people through the justice system [29, 41, 49–51, 53, 56, 57, 59, 73, 80, 82], specifically for or customised to meet the needs of the community they served [32, 47, 84, 88]. • providing advice in relation to **public health initiatives** not within the normal scope of mainstream PHC including sanitation system construction and maintenance, disease surveillance, environmental health, food distribution, and transportation [59]. • **collaborating with other organisations** such as schools, youth groups, prisons, disability and aged care services [29, 32, 81], and with councils, liquor outlets and grocers to reduce the supply of harmful products while increasing the availability of healthy options [29, 36, 47, 61].
**Self-determination and Empowerment** • facilitating **self-determination and empowerment** of Indigenous communities in order to establish and manage their own Indigenous health services [30, 32], enabling clients to take control of their own health, at an individual and family level [69], building resilience [53], and enabling empowerment of clients through program engagement [34, 40, 47, 59, 74, 77, 83, 87]. • providing **employment and training** to promote the development of the local Indigenous health workforce, and build the capacity of the community [32, 40, 41, 87]. • facilitating **leadership opportunities** to ensure that Indigenous health care staff take on leadership roles within their communities [32] and provide positive role models for other Indigenous peoples [32, 70]. • promoting **community development** through the organisation of activities beyond health care [36, 49], such as cultural days and camps, reconciliation events and other community activities enable communities to draw on culture, increase social connectedness, and have pride in their identity [41].

While each of these characteristics were clearly identifiable as independent themes within the literature, the interdependence between characteristics was also evident. As mentioned, culture was interwoven throughout the seven other characteristics. Culture facilitated assessable health services, informing the delivery of culturally appropriate services by making them acceptable by the community [34, 47, 54, 58, 74, 80, 82, 87]. Culture was critical to ensuring community participation, enabling Indigenous ownership and governance by engaging communities [30–32, 34, 35, 40, 60, 61, 64, 70, 82], and engaging in quality improvement process and defining outcomes and indicators [64]. Culture was important in ensuring the approach to care is culturally appropriate and relevant [36, 82, 83], and holistic including comprehensive and providing a diverse range of care, which included traditional healing [36, 52, 59, 63, 64, 70, 72, 73, 77, 79–82, 84–88]. Culture informed and supported the philosophy underpinning Indigenous self-determination, particularly community participation, ensuring Indigenous peoples having the right and determination to decide how their PHC services should, and can be delivered [30, 33, 40, 49, 59, 69, 73, 82, 85].

Another example of interdependence between characteristics is culturally appropriate and skilled workforce, which was an enabler for culture, holistic health care and accessible health services. Employing local Indigenous staff helped to embed community cultural values, customs and beliefs into service delivery [45, 77, 78, 81]. Workforce was central to the delivery of services, providing a holistic comprehensive PHC and a diverse range of care [38, 52, 83, 85, 87, 88]. A culturally appropriate and skilled workforce also enabled services to be accessible and acceptable, by building trust with communities [45, 83, 87], ensuring patients felt supported [38, 41], providing assurances in relation to privacy and confidentiality [38, 80], and implementing services underpinned by cultural respect, social justice and equality [47].

**Fig. 2:**
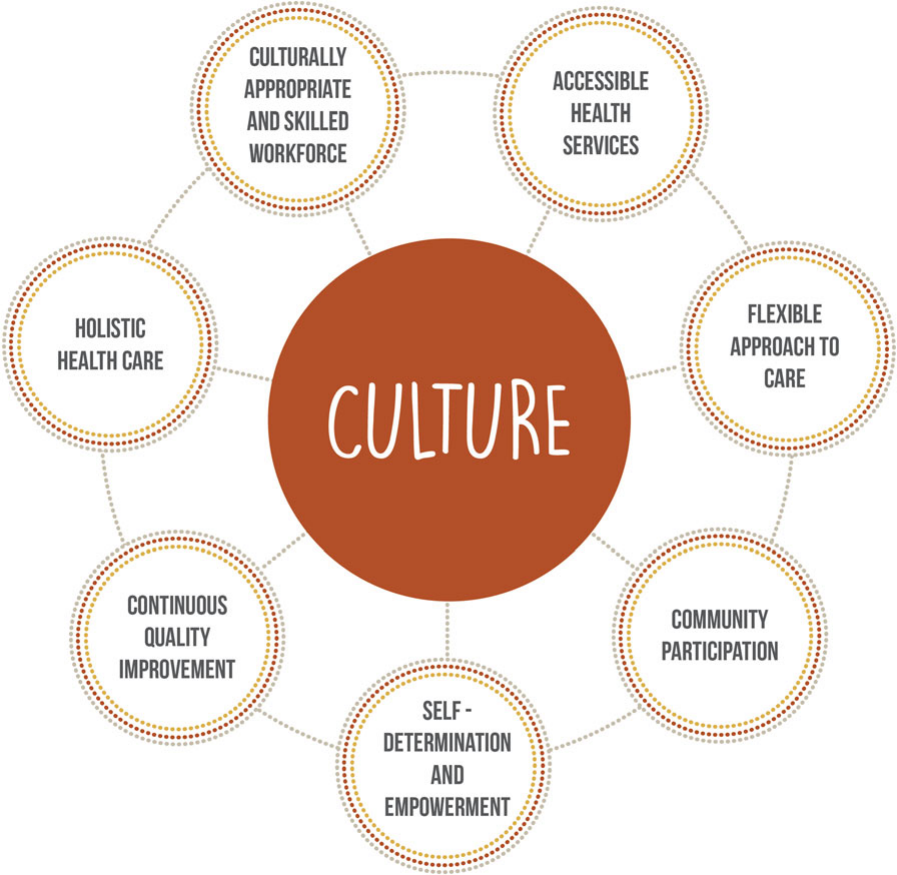
Characteristics of Indigenous Primary Health Care Service Delivery Model

As a result of identifying the characteristics of Indigenous PHC service delivery models, we have identified and described a new Indigenous PHC Service Delivery Model, as depicted in Fig. 2. Fundamental to this model is culture, as previously discussed culture plays a central role in Indigenous PHC service delivery models; and is encompassed by the seven other characteristics – accessible health services, community participation, continuous quality improvement, culturally appropriate and skilled workforce, flexible approach to care, holistic health care, and self-determination and empowerment. While Table 1 provides details as to how the characteristics have been embedded throughout other Indigenous PHC service delivery models, they also act as examples of how the characteristics of the Indigenous PHC Service Delivery Model can be implemented within other services.

## Discussion

The aim of this scoping review was to identify the characteristics of Indigenous PHC service delivery models. We found that culture underpinned all aspects of the Indigenous PHC service delivery models identified in this review. In addition, we identified seven other distinct characteristics of Indigenous PHC service delivery models – accessible health services, community participation, continuous quality improvement, culturally appropriate and skilled workforce, flexible approach to care, holistic health care, and self-determination and empowerment.

These findings suggest that Indigenous PHC service delivery models are somewhat different to many of the models of care developed within western contexts. For example, the *Chronic Care Model* [90] focuses on implementing evidence based care, mobilising community resources, enabling patient’s self-management, and ensuring coordinated care and health promotion. The explicit role of culture in the provision of services is notably absent from this model. Culture is also notably absent from the *World Health Organisation Innovative Care for Chronic Conditions Framework* [91] and the *Southgate Model of Comprehensive Primary Health Care in Australia* model [92].

By contrast, it is evident that local cultural values, customs and beliefs were at the centre of and underpinned all aspects of care in Indigenous PHC service delivery models. This was a common thread in the majority of the studies included in this scoping review. The role of culture as a defining characteristic, therefore, provides the greatest distinction between Indigenous PHC service delivery and other models of care. This is consistent with the growing literature on culture and health, which describes the importance of culture and its effect on health and wellbeing [2, 93–95], including in non-Indigenous populations [96]. These are potential lessons mainstream health care services could learn from in order to make their services more culturally safe and appropriate to Indigenous peoples. Three characteristics stand out as distinctive aspects of Indigenous PHC service delivery models. These are culturally appropriate and skilled workforce; community participation; and self-determination and empowerment. At the heart of a culturally appropriate and skilled workforce were the Indigenous staff. This supports the belief that Indigenous PHC services are best delivered by Indigenous peoples [17]. In particular, employing local Indigenous staff helps to embed the community’s cultural values, customs and beliefs into service delivery. Indigenous peoples providing health care to their own people has been shown to improve health outcomes related to diabetes [97], asthma [98], mental health and maternal and infant care [99]. Importantly, local Indigenous staff provide more acceptable care [100], as well as encouraging access to PHC more generally [101]. One of the many challenges faced by Indigenous PHC services is the need to maintain current levels of Indigenous staff, while at the same time, growing their Indigenous health workforce [66, 80]. This requires a partnership between Indigenous PHC services and governments to ensure the growth of the sector is done in a way that is meaningful and culturally safe.

Community participation was found to be particularly important for ensuring Indigenous PHC services continue to identify, understand and address the needs of local Indigenous peoples. Community participation also facilitated Indigenous governance and ownership. One example is the Southcentral Foundation in Anchorage Alaska, it is possibly one of the most well-known Indigenous models of care (four of the included studies are from the Southcentral foundation [60, 64, 69, 76]). The success of this model, including significant improvements in health outcomes is associated with the notion of ‘customer-owners’ ([102] p. 1). The model ensures that Alaska Native people are in control of their health service and the relationship that is built and maintained by the service with its ‘customer-owners’ ([102] p. 1). Many of these services included in this review were also underpinned by the philosophy that Indigenous peoples having the right to decide how their PHC services should be developed and delivered. Aboriginal Community Controlled Health Organisations in Australia have been identified as exemplars of these types of community governance models [103], further demonstrating the link between community control and positive health outcomes.

Self-determination and empowerment were the driving principles behind the establishment of many Indigenous PHC services included in this review. Indigenous PHC services facilitated a number of opportunities for self-determination and empowerment. These include ensuring that Indigenous peoples are able to take control of their own health service; the employment and training of Indigenous peoples; and just as importantly community development initiatives such as cultural days and camps, and reconciliation events. Previous studies have demonstrated an association between empowerment of Indigenous peoples and communities and better health outcomes for Indigenous peoples [104, 105]. As on example, a systematic review conducted by Minichiello et al. [106], found that tobacco intervention programs which had elements of self-determination and were relevant to the community were more likely to lead to positive outcomes such as reduced initiation and consumption of tobacco. Often self-determination and empowerment are associated with community participation. Community participation and mobilization in health care is essential for ensuring services can identify health needs and set priorities, plan, implement, monitor and evaluate services and programs, this is consistent with existing literature on community participation in health [107].

Indigenous PHC service delivery models are exceptional models of PHC, delivering health care to Indigenous peoples and communities across the globe, often to isolated populations or communities where no other service delivery model is viable. While there are numerous benefits to Indigenous PHC service delivery models, there are also limitations: Indigenous PHC service delivery models do not align with government funding mechanism [108, 109]; there is often a lack of funding to support specific Indigenous PHC services [108–110]; the need for services outweighs funding and the availability of services [108–110]; and the delivery of health care to Indigenous peoples is more expensive [108, 109].

Policies need to acknowledge and take into account the differences between Indigenous PHC models of care and other PHC models of care. This is particularly crucial when it comes to the funding of Indigenous PHC services, as the lack of sufficient funding together with the uncertainty that comes from short-term funding models led to the inability of services included in this review to support the unique characteristics of Indigenous PHC services [27, 32, 33, 40, 51, 54, 78, 82]. Funding models are one of the key drivers for how care is provided [111]. Yet it is also the case that until Indigenous PHC service delivery models are clearly articulated, policy makers will not be able to design appropriate funding mechanisms to support the way in which they provide care. We believe that the Indigenous PHC service delivery characteristics identified by this scoping review is one step towards making Indigenous PHC service delivery models explicit.

While this is the first review of its type to identify the characteristics of Indigenous PHC service delivery models, a review by Lewis and Myhra on *Integrated care with Indigenous populations: a systematic review of the literature* [112], was identified. However, that review focused on assessing how health care services are conceptualising and enacting integrated care with American Indian and First Nations populations in the United States and Canada and the successes and challenges of carrying out these interventions with this population. It identified the motivations for integration and its effectiveness. Our review was more inclusive of health services and Indigenous populations from across the global and focused on the characteristics of Indigenous PHC service delivery models rather than the motivations of one particular service delivery model.

## Conclusion

Indigenous PHC services evolved as a result of mainstream health services inability to meet the needs of Indigenous peoples [3, 7, 8] and Indigenous peoples often being excluded and marginalised from mainstream health services [9]. In addition, Indigenous communities wanted to be able to provide care to their communities that is culturally appropriate, comprehensive, holistic, accessible, and community controlled. The review identified eight characteristics of Indigenous PHC service delivery models. These characteristics were found to be global in their application, and provide insight and guidance to communities and organisations wishing to initiate an Indigenous PHC service and programs. The review also affirms and supports the philosophy underpinning Indigenous self-determination, particularly Indigenous peoples having the right and determination to decide how their PHC services should be and can be delivered for themselves. If communities and governments are genuinely serious about closing the gaps in life expectancy and morbidity between Indigenous and non-Indigenous peoples, then Indigenous PHC services must continue to be supported.

## Additional files

Additional file 1: Search strategy. (DOCX 13 kb)

Additional file 2: Included studies. (DOCX 16 kb)

## Abbreviations

PHC: Primary Health Care
