# Supplementary material for: Characteristics of Indigenous primary health care service delivery models: a systematic scoping review
Source: Global Health. 2018 Jan 25;14:12. doi: 10.1186/s12992-018-0332-2 (PMC5784701; doi:10.1186/s12992-018-0332-2)
Supplement: Supplementary file 1 — Search strategy. (DOCX 13 kb) [file 12992_2018_332_MOESM1_ESM.docx]

**Additional File 1: Search Strategy**

PubMed

| Number | Search | Results |
| --- | --- | --- |
| #1 | Aborigin*[tiab] OR indigenous[tiab] OR first nation[tiab] OR native[tiab] OR Inuit[tiab] OR Metis[tiab] OR American Indian[tiab] OR Maori[tiab] OR indigenous populations[MeSH Terms] OR australian aborigine[MeSH Terms] OR native american[MeSH Terms] OR american indian[MeSH Terms] OR oceanic ancestry group[MeSH Terms] OR inuit[MeSH Terms] OR american native continental ancestry group[MeSH Terms] | 389,225 |
| #2 | Primary health care[tiab] OR Primary healthcare[tiab] OR primary health-care[tiab] OR comprehensive primary healthcare[tiab] OR comprehensive primary health care[tiab] OR medical service[tiab] OR health service[tiab] OR community care[tiab] OR community health service[tiab] OR primary health care[MeSH Terms] OR comprehensive health care[MeSH Terms] OR community health service[MeSH Terms] | 710,568 |
| #3 | model[tiab] | 1,410,425 |
| #4 | #1 AND #2 AND #3 | 1,896 |

(((Aborigin*[tiab] OR indigenous[tiab] OR first nation[tiab] OR native[tiab] OR Inuit[tiab] OR Metis[tiab] OR American Indian[tiab] OR Maori[tiab] OR indigenous populations[MeSH Terms] OR australian aborigine[MeSH Terms] OR native american[MeSH Terms] OR american indian[MeSH Terms] OR oceanic ancestry group[MeSH Terms] OR inuit[MeSH Terms] OR american native continental ancestry group[MeSH Terms])) AND (Primary health care[tiab] OR Primary healthcare[tiab] OR primary health-care[tiab] OR comprehensive primary healthcare[tiab] OR comprehensive primary health care[tiab] OR medical service[tiab] OR health service[tiab] OR community care[tiab] OR community health service[tiab] OR primary health care[MeSH Terms] OR comprehensive health care[MeSH Terms] OR community health service[MeSH Terms])) AND (model[tiab])
