# Supplementary material for: Characteristics of Indigenous primary health care service delivery models: a systematic scoping review
Source: Global Health. 2018 Jan 25;14:12. doi: 10.1186/s12992-018-0332-2 (PMC5784701; doi:10.1186/s12992-018-0332-2)
Supplement: Supplementary file 2 — Included studies. (DOCX 16 kb) [file 12992_2018_332_MOESM2_ESM.docx]

Additional File 2: Included Studies

| **First author, date**  **Published** | **State and Country** | **Focus of service delivery model** | **Setting** |
| --- | --- | --- | --- |
| 2002 | Queensland, Australia | Women’s health | Rural |
| Allison 2007 | Arizona, US | Public health | Rural |
| Arora 2013 | Alberta, Canada | Eye health | Remote |
| Auclair 2012 | Nunavik, Canada | Mental health | Remote |
| Bailie 2013 | Australia | Continuous quality improvement | Mixed |
| Baldwin 2001 | Alaska, US | Maternal health | Remote |
| Bartlett 2001 | Northern Territory, Australia | General health | Remote |
| Bennett 1988 | Victoria, Australia | General health | Urban |
| Benoit 2003 | British Columbia, Canada | Women’s health | Urban |
| Berner 1992 | Alaska, US | General health & Indigenous health worker | Remote |
| Birks 2010 | Queensland, Australia | General health | Remote |
| Bowyer 1997 | Minnesota, US | Eye health | Rural |
| Brant 1999 | Montana, and Wyoming, US | Women’s health & prevention and health promotion | Mixed |
| Campbell 1995 | New South Wales, Australia | General health | Urban |
| Campbell 2015 | New South Wales, Australia | Oral health | Mixed |
| Central Australia Aboriginal Congress | Northern Territory, Australia | General health | Rural |
| Centre for Primary Care 2015 | Alaska, US | General health | Urban |
| Chang 2000 | Australia | Asthma | Very remote |
| Chiarchiaro 1997 | Oklahoma, US | Oral health | Urban |
| Coll 2004 | Alaska, Michigan, South Dakota, US | General health | Mixed |
| Copeman 1988 | Australia | General health |  |
| Crengle 2000 | New Zealand | General health |  |
| Davy 2012 | Papua New Guinea | General health | Rural |
| Dawson 2003 | South Australia, Australia | Asthma | Rural |
| Dietrich 1986 | New Mexico, US | General health | Rural |
| DiGiacomo 2010 | New South Wales, Australia | Adult health | Urban |
| Driscoll 2013 | Alaska, US | General health | Mixed |
| Dyson 2012 | Western Australia, Australia | Oral health | Mixed |
| Eby 2007 | Alaska, US | General health | Urban |
| Freeman 2014a | Northern Territory, and South Australia, Australia | General health | Mixed |
| Freeman 2014b | Northern Territory, and South Australia, Australia | General health | Mixed |
| Gabrysch 2009 | Huamanga, Peru | Maternal health | Rural |
| Gajjar 2014 | Queensland, Australia | General health | Urban |
| Gardner 2010 | New South Wales, Northern Territory, Queensland, and Western Australia, Australia | Continuous quality improvement | Mixed |
| Janssen 2014 | Marlborough, New Zealand | Diabetes | Urban |
| Johnston 2013 | Alaska, US | Homecare and outreach | Mixed |
| Kahn 1988 | Arizona, US | Mental health | Rural |
| Kelaher 2006 | Australia | Medicine | Remote |
| Kelly 2007 | South Australia, Australia | Women’s health & sexual health | Urban |
| Langwell 2014 | Wyoming, US | General health | Remote |
| Lantz 2003 | Alaska, South Dakota, Oklahoma, and Washington, US | Prevention and health promotion & cancer | Mixed |
| Lovett 2014 | Australian Capital Territory, Australia | Alcohol and other drugs | Urban |
| Lyford 2005 | Bay of Plenty, New Zealand | General health | Urban |
| Maar 2009 | Ontario, Canada | Mental health | Rural |
| Maniapoto 2003 | Auckland, New Zealand | General health | Urban |
| Murphy 2012 | New South Wales, Australia | Maternal health | Mixed |
| Nebelkopf 2003 | California, US | General health | Urban |
| Panarettp 2005 | Queensland, Australia | Maternal health | Rural |
| Panaretto 2014 | Queensland, Australia | General health | Mixed |
| Pelcastre-Villafuerte 2014 | Chiapas, Guerrero, Oaxaca, and Puebla, Mexico | Women’s health | Mixed |
| Poroch 2007 | Australian Capital Territory, Australia | Prison health | Urban |
| Poroch 2012 | Australian Capital Territory, Australia | Prison health | Urban |
| Reeve 2015 | Western Australia, Australia | General health | Remote |
| Smith 2000 | US | Prevention and health promotion |  |
| Stewart 2009 | Queensland, Australia | General health | Rural |
| Taylor 2001 | Australia | General health |  |
| Tongs 2007 | Australian Capital Territory, Australia | Prison health | Urban |
| Tongs 2014 | Australian Capital Territory, Australia | General health | Urban |
| Townsville Aboriginal Islanders Health Service 2003 | Queensland Australia | Maternal and infant health | Rural |
| Tyree 2007 | North Dakota and South Dakota, US | General health & Indigenous health worker | Rural |
| Wakerman 1998 | Northern Territory, Australia | General health | Remote |
| West 1993 | US | General health |  |
